# Effects of time delay and body temperature on measurements of central venous oxygen saturation, venous-arterial blood carbon dioxide partial pressures difference, venous-arterial blood carbon dioxide partial pressures difference/arterial-venous oxygen difference ratio and lactate

**DOI:** 10.1186/s12871-018-0655-9

**Published:** 2018-12-11

**Authors:** Xiang-yu Wan, Li-li Wei, Yan Jiang, Ping Li, Bo Yao

**Affiliations:** 1 Department of Critical Care Medicine, the Affiliated Hospital of Qingdao University, Jiangsu Road 16, Qingdao, 266000 China; 2 Department of Nursing, the Affiliated Hospital of Qingdao University, Jiangsu Road 16, Qingdao, 266000 China; 3 Department of Critical Care Medicine, the Affiliated Hospital of Qingdao University, Haier Road 16, Qingdao, 266000 China

**Keywords:** ScvO_2_, Pv-aCO_2_, Lactate, Pv-aCO_2_/Ca-vO_2_, Time delay, Body temperature

## Abstract

**Background:**

Central venous oxygen saturation (ScvO_2_), venous-arterial blood carbon dioxide partial pressures difference (Pv-aCO_2_), venous-arterial blood carbon dioxide partial pressures difference/arterial-venous oxygen difference ratio (Pv-aCO_2_/Ca-vO_2_) and lactate are important parameters employed during shock resuscitation. We designed this study to confirm the effects of time delay and body temperature on measurements of these four parameters.

**Methods:**

Arterial and central venous blood samples were simultaneously drawn by plastic syringes via indwelling intra-arterial and central venous catheters from critically ill patients. Blood gas analyses were performed on both samples and repeated after 10, 20, 30, 40, 50 and 60 min. Patients were divided into a control group and a high temperature group according to whether the body temperature was greater than 38 °C.

**Results:**

A total of 30 critically ill patients were enrolled. There was a trend of increasing values for ScvO_2_, Pv-aCO_2_, Pv-aCO_2_/Ca-vO_2_ and lactate over time (*P* < 0.001). The ScvO_2_ differences were all lower in high temperature group after 10, 20, 30, 40, 50 and 60 min when compared to the corresponding differences in the control group (*P* < 0.05). The differences in lactate values were slightly higher in the high temperature group, relative to the control group after 20, 30, 40, 50 and 60 min (*P* < 0.05).

**Conclusions:**

Measurements of ScvO_2_, Pv-aCO_2_, lactate and Pv-aCO_2_/Ca-vO_2_ were affected by time delay or body temperature. We recommend that arterial and central venous blood gas samples be analyzed quickly within 10 min, especially for patients with body temperature <38 °C.

**Trial registration:**

ChiCTR, ChiCTR1800014484. Registered 16 January 2018.

## Background

Early recognition and accurate correction of tissue hypoperfusion are important in the treatment of shock. Central venous oxygen saturation (ScvO_2_), venous and arterial blood carbon dioxide partial pressure difference (Pv-aCO_2_), the venous–arterial blood carbon dioxide partial pressure difference/arterial-venous oxygen content difference ratio (Pv-aCO_2_/Ca-vO_2_) and lactate are important parameters employed during shock resuscitation [1]. ScvO_2_ is associated with global tissue oxygenation, which can reflect the global balance between arterial O_2_ delivery and O_2_ consumption. If the value is low, anaerobic metabolism may arise. It was proved useful for hemodynamic management [2], and was recommended as one of the early resuscitation goals in 2012 sepsis guidelines [3]. Although ScvO_2_ was deleted in early resuscitation goals in 2016 sepsis guidelines [4], it is still a useful clinical parameter. A ΔScvO_2_ threshold value of 5.0% discriminated fluid responders from non-responders with a sensitivity of 0.78 and specificity of 0.95 [5]. Moreover, ScvO_2_ can also predict the success of spontaneous breathing trial [6]. Pv-aCO_2_ is another alternative possible marker of tissue perfusion. According to the Fick equation, Pv-aCO_2_ is inversely related to cardiac output [7]. A normal Pv-aCO_2_<5 mmHg can be used as an adjunctive hemodynamic parameter to exclude tissue hypoperfusion in patients undergoing major surgery [8]. Pv-aCO_2_ can also help clinicians make decisions of regarding the provision of therapy aimed at increasing cardiac output [7]. The Pv-aCO_2_/Ca-vO_2_ ratio was shown to be a good indicator of anerobiosis assessed by hyperlactatemia. Mekontso-Dessap A et al. found that the Pv-aCO_2_/Ca-vO_2_ ratio (cut-off value ≥1.6) can detect global anaerobic metabolism in critically ill patients [9]. The Pv-aCO_2_/Ca-vO_2_ ratio may provide useful information for assessing the lactate clearance potential and optimizing the lactate clearance rate [10]. Moreover, the Pv-aCO_2_/Ca-vO_2_ ratio (cut-off value≥1.6) was an independent predictor of ICU mortality in septic shock patients with high ScvO_2_ after resuscitation [11]. Increased serum lactate is an early indicator of septic shock and helps accomplish resuscitation goal, and it is recommended utilizing in sepsis guidelines [3, 4]. Given these four parameters above, and their values in shock resuscitation, some researchers have compiled them into a flow sheet to guide resuscitation therapy in septic shock [1].

Before using a clinical parameter in therapeutic decisions, its accuracy is important. Unfortunately, previous studies have found that the values of arterial oxygen partial pressure (PaO_2_) and arterial carbon dioxide partial pressure (PaCO_2_) were influenced by time delay and sample storage temperature in blood gas analysis [12, 13]. The effects of time delay and body temperature on the measurements of ScvO_2_, Pv-aCO_2_, lactate and Pv-aCO_2_/Ca-vO_2_ have not been adequately studied. Thus, we designed this study to confirm the effects of time delay and body temperature on these four specific parameters.

## Methods

The study was approved by the Ethics Committee of our hospital and registered at Chinese clinical trial registry (ChiCTR1800014484). It was performed from January 2018 to May 2018 in the intensive care unit of the affiliated hospital of Qingdao university. The patients with indwelling central venous and intra-arterial catheters were enrolled. Therefore, no additional invasive procedures were performed on patients merely to collect blood samples. Patients treated with antipyretic drugs or physical cooling were excluded.

A 2 mL sample of arterial blood was drawn from the indwelling intra-arterial catheter, and simultaneously, 2 mL venous blood sample was collected from the central venous catheter using plastic blood gas syringes (BD Preset™3ml, 22G × 1″, Becton, Dickinson and Company, United Kingdom) by a nurse. The nurse repeatedly drew blood in this manner for a total of 7 times, producing 7 sets of central venous and arterial blood samples simultaneously. Blood gas analysis was performed on one of the sets of the venous and arterial samples via blood gas analyzer (Radiometer ABL90 FLEX, Denmark) as soon as possible after sampling by another nurse. The time needed for transporting samples to the blood gas analyzer was less than 2 min for the first analysis. Then, blood gas analyses were repeated at 10, 20, 30, 40, 50 and 60 min after drawing for remaining 6 sets of samples. During the time delay, the blood samples were stored at 22 °C (room temperature). Pv-aCO_2_ and the Pv-aCO_2_/Ca-vO_2_ ratio were calculated [11].

The patients’ baseline data were also collected, such as age, gender, Acute Physiology and Chronic Health Evaluation (APACHE) II scores and primary diseases. Patients were divided into either the control group or the high temperature group according to whether their body temperature was greater than 38 °C. Body peripheral temperature was measured by mercury thermometer in axilla. Drawing blood gas samples and measuring body temperature were performed simultaneously.

### Statistics analysis

The statistical analysis was performed using SPSS 22.0 software (SPSS, Inc., Chicago, IL, USA). The results were expressed as mean ± SD. Statistical analysis of the parameters over time was analyzed by repeated-measures ANOVA (analysis of variance). The statistical significance was set at *P* <0.05.

## Results

A total of 30 critically ill patients were enrolled. The baseline characteristics of them are shown in Tables 1 and 2. There were no significant differences in baseline data between two groups (*P* > 0.05).

**Table 1:** Baseline characteristics of patients

Variables	Value
Age (years)	63 ± 16
Gender (male/female)	19/11
APACHE II scores	21 ± 5
Hemoglobin (g/L)	96.3 ± 26.4
White blood cells (10^9^/L)	15.7 ± 11.2
Primary diseases	
Pneumonia	12
Abdominal infection	8
Gastrointestinal hemorrhage	5
Heart failure	4
Bacterial liver abscess	1

**Table 2:** Baseline characteristics of two groups

Variables	Control group (*n* = 19)	High temperature group (*n* = 11)	*P* value
Age (years)	62 ± 17	65 ± 13	0.704
Gender (male/female)	11/8	8/3	0.466
APACHE II scores	21 ± 6	20 ± 5	0.770
Hemoglobin (g/L)	91.9 ± 19.2	103.9 ± 35.5	0.320
White blood cells (10^9^/L)	14.9 ± 8.5	17.2 ± 15.1	0.597
Catecholamine therapy (yes/no)	11/8	6/5	0.858
Sepsis (yes/no)	9/10	7/4	0.389
Surgery (yes/no)	10/9	4/7	0.389
Trauma (yes/no)	1/18	0/11	1.000
PO_2_ baseline (mmHg)	123 ± 106	107 ± 34	0.551
ScvO_2_ baseline (mmHg)	67.7 ± 9.4	72.8 ± 7.7	0.140
Patmospheric-vO_2_ baseline (mmHg)	122.2 ± 5.9	111.9 ± 5.8	0.084
PvCO_2_ baseline (mmHg)	47.7 ± 11.2	45.2 ± 11.3	0.413
PaCO_2_ baseline (mmHg)	42.6 ± 12.1	39.1 ± 10.1	0.435
Pv-aCO_2_ baseline (mmHg)	6.1 ± 2.5	6.0 ± 2.4	0.920
Pv-aCO_2_/Ca-vO_2_ baseline	1.7 ± 0.6	1.8 ± 0.5	0.626
Lactate (mmol/L)	1.4 ± 0.8	2.4 ± 2.1	0.149

There was a trend of increasing values for ScvO_2_, Pv-aCO_2_, Pv-aCO_2_/Ca-vO_2_ and lactate over time (*P* < 0.001). ScvO_2_, Pv-aCO_2_/Ca-vO_2_ and lactate values of the samples stored for 10, 20, 30, 40, 50 and 60 min were significantly higher relative to baseline (*P* < 0.05). There were no significant differences of PaCO_2_ and PvCO_2_ value over time (*P* > 0.05). The values for Pv-aCO_2_ only increased after 40, 50 and 60 min compared to baseline (*P* < 0.05), but there were no significant differences after 10, 20 or 30 min (Table 3).

**Table 3:** Changes in values at different time points

Variables	Baseline	10 min	20 min	30 min	40 min	50 min	60 min
ScvO_2_ (%)	69.6 ± 9.0	71.8 ± 8.3^*^	73.8 ± 8.3^*^	75.9 ± 8.0^*^	77.5 ± 7.8^*^	79.6 ± 8.1^*^	81.7 ± 7.5^*^
PaCO_2_ (mmHg)	41.4 ± 11.4	41.7 ± 11.3	42.2 ± 11.4	42.5 ± 11.4	42.9 ± 11.3	43.2 ± 11.2	43.6 ± 11.1
PvCO_2_ (mmHg)	47.4 ± 11.2	47.8 ± 10.9	48.3 ± 10.7	48.9 ± 10.7	49.4 ± 10.9	49.9 ± 10.7	50.3 ± 10.5
Pv-aCO_2_ (mmHg)	6.1 ± 2.4	6.1 ± 2.5	6.1 ± 2.5	6.3 ± 2.5	6.6 ± 2.6^*^	6.7 ± 2.8^*^	6.7 ± 2.9^*^
Pv-aCO_2_/Ca-vO_2_	1.7 ± 0.6	1.9 ± 0.6^*^	2.1 ± 0.8^*^	2.6 ± 1.6^*^	3.0 ± 1.9^*^	3.3 ± 2.2^*^	4.0 ± 3.5^*^
Lactate (mmol/L)	1.8 ± 1.5	1.9 ± 1.5^*^	2.1 ± 1.5^*^	2.2 ± 1.5^*^	2.3 ± 1.5^*^	2.4 ± 1.6^*^	2.6 ± 1.6^*^

^*^significant difference compared to baseline (*P* <0.05)

There was a trend of increasing ScvO_2_ differences, Pv-aCO_2_ differences, Pv-aCO_2_/Ca-vO_2_ differences and lactate differences between baseline and some point along storage time (*P* < 0.05). The ScvO_2_ differences between baseline and after 10, 20, 30, 40, 50 and 60 min were all lower in the high temperature group than differences in the control group (*P* < 0.05). The lactate differences between baseline and 20, 30, 40, 50 and 60 min were all slightly higher in the high temperature group than the differences in the control group (P < 0.05). Pv-aCO_2_/Ca-vO_2_ ratio differences between baseline and 10 min were lower in the high temperature group than the ratio differences in the control group (*P* < 0.05) (Table 4).

**Table 4:** Comparisons of values between two groups

Groups	10 min	20 min	30 min	40 min	50 min	60 min
△ScvO_2_						
Control group (*n* = 19)	2.8 ± 1.5	4.9 ± 1.6	7.2 ± 2.7	9.1 ± 3.2	11.4 ± 3.5	14.0 ± 4.4
High temperature group (*n* = 11)	1.4 ± 0.9	3.2 ± 1.7	4.9 ± 1.8	6.1 ± 2.5	7.5 ± 3.4	8.9 ± 4.3
P value	0.013	0.014	0.016	0.011	0.006	0.005
△Pv-aCO_2_						
Control group (*n* = 19)	0.1 ± 0.6	0.1 ± 1.0	0.3 ± 1.0	0.7 ± 0.8	0.7 ± 0.9	0.8 ± 1.1
High temperature group (*n* = 11)	−0.2 ± 0.4	−0.2 ± 0.9	0.2 ± 0.8	0.1 ± 1.0	0.4 ± 1.2	0.4 ± 1.4
P value	0.138	0.469	0.668	0.054	0.437	0.313
△Pv-aCO_2_/ Ca-vO_2_						
Control group (*n* = 19)	0.23 ± 0.17	0.46 ± 0.49	1.1 ± 1.7	1.51 ± 1.94	1.89 ± 2.13	2.79 ± 3.85
High temperature group (*n* = 11)	0.04 ± 0.16	0.25 ± 0.39	0.59 ± 0.63	0.77 ± 1.04	1.10 ± 1.30	1.41 ± 1.46
P value	0.005	0.252	0.374	0.253	0.279	0.264
△Lactate						
Control group (*n* = 19)	0.13 ± 0.08	0.23 ± 0.10	0.33 ± 0.12	0.43 ± 0.18	0.51 ± 0.19	0.62 ± 0.22
High temperature group (*n* = 11)	0.16 ± 0.08	0.35 ± 0.14	0.51 ± 0.31	0.72 ± 0.44	0.89 ± 0.62	1.14 ± 0.80
P value	0.309	0.012	0.030	0.015	0.016	0.012

*△ScvO*_*2*_ *x min* ScvO_2_ x min- ScvO_2_ baseline

*△Pv-aCO*_*2*_ *x min* Pv-aCO_2_ x min- Pv-aCO_2_ baseline

*△Pv-aCO*_*2*_*/ Ca-vO*_*2*_ *x min* Pv-aCO_2_/ Ca-vO_2_ x min- Pv-aCO_2_/ Ca-vO_2_ baseline

*△Lactate x min* Lactate x min- Lactate baseline

## Discussion

In this study, we found that there was a trend of increasing values for ScvO_2_, Pv-aCO_2_, Pv-aCO_2_/Ca-vO_2_ and lactate values with increased storage time. The differences in ScvO_2_, Pv-aCO_2_/Ca-vO_2_ and lactate between certain periods of storage time and baseline demonstrated differences between the high temperature group and the control group along certain points of storage time.

According to this study, we often over-estimated SvO_2_ levels of the central venous blood samples. The guideline of the American Association for Respiratory Care states that a delay in analysis of a venous blood gas of 30 min is acceptable [14]. After a 30 min time delay in analysis, the ScvO_2_ increased approximately 6% in our study. This indicated that the guideline may lead to unsafe practices. The ScvO_2_ value was increasing over all storage times in the study, so we recommend blood gas samples be analyzed as soon as possible. It was proven that the PO_2_ of blood stored in a glass syringe decreases slightly over time, primarily as a result of continuing cellular metabolism [13]. However, plastic syringes have supplanted glass syringes because of their convenience, low cost and breakage resistance. But plastic syringes, unlike glass syringes, are permeable to O_2_ [15]. Atmospheric O_2_ has a partial pressure of 159 mmHg, well above venous blood O_2_. Therefore, O_2_ will permeate into plastic syringes until there is O_2_ partial pressure equilibration between the blood sample and the atmosphere. A previous study also indicated that the ScvO_2_ value increased over storage time [16]. In this study, we found that the rate of ScvO_2_ change in patients with a temperature ≥ 38 °C was lower than patients with temperature< 38 °C. The baseline ScvO_2_ values and atmospheric-venous blood O_2_ partial pressure difference between two groups displayed no difference. Therefore, the increasing O_2_ consumption may be explained the effects of temperature on ScvO_2_. We speculated that blood cells have an intrinsically hypermetabolic state in febrile patients and subsequently consumed more O_2_. Lactate is one of metabolites. The faster rising lactate in the high temperature group of this study may support the idea that blood cells in febrile patients had a high hypermetabolic state. Advanced cell experiments were needed to confirm the hypothesis. In previous studies, temperature correction of blood gas samples is not recommended [14, 17]. But according to our results, the effect of temperature on ScvO_2_ should not be ignored.

There were no significant differences of PaCO_2_ and PvCO_2_ value over time. The Pv-aCO_2_ increased only approximately 0.2 mmHg after a 30 min time delay in analysis, which may not have very important clinical implications. Furthermore, no effect of temperature on Pv-aCO_2_ was found. So there were few clinical effects of time delay and temperature on Pv-aCO_2_ value. In the calculation of Pv-aCO_2_/Ca-vO_2_ [11], O_2_ partial pressure is an important parameter. Because of effects of the time delay on blood O_2_ partial pressure [12, 13], Pv-aCO_2_/Ca-vO_2_ may also be affected by time delay. In actuality, our results proved that this is the case. After a 30 min time delay, the ratio increased 1 by almost. Further, Pv-aCO_2_/Ca-vO_2_ difference was lower in patients in the high temperature group. At 10 min, the value demonstrated a significant difference. Lactate values were also affected by time delay and temperature, but the influence was not very strong. After a 30 min time delay, the lactate increased only approximately 0.4 mmol/L.

## Conclusions

There were some influences of time delay or body temperature on ScvO_2_, Pv-aCO_2_, lactate and Pv-aCO_2_/Ca-vO_2_ values. We recommend that arterial and central venous blood gas samples be analyzed quickly within 10 min, especially for patients with body temperature <38 °C.

## Abbreviations

APACHE: Acute Physiology and Chronic Health Evaluation
Lac: Lactate
PaCO_2_: Arterial carbon dioxide partial pressure
PaO_2_: Arterial oxygen partial pressure
Pv-aCO_2_: Venous and arterial blood carbon dioxide partial pressures difference
Pv-aCO_2_/Ca-vO_2_: Venous and arterial blood carbon dioxide partial pressures difference/arterial-venous oxygen difference ratio
ScvO_2_: Central venous oxygen saturation
